# MicroRNA-3196 is inhibited by H2AX phosphorylation and attenuates lung cancer cell apoptosis by downregulating PUMA

**DOI:** 10.18632/oncotarget.12794

**Published:** 2016-10-21

**Authors:** Chengshan Xu, Ling Zhang, Lianning Duan, Chengrong Lu

**Affiliations:** ^1^ Aviation Medicine Research Laboratory, Air Force General Hospital, PLA, Beijing 100142, China; ^2^ Key Laboratory of RNA Biology, Institute of Biophysics, Chinese Academy of Sciences, Beijing 100101, China

**Keywords:** lung cancer, histone H2AX, miR-3196, PUMA, apoptosis

## Abstract

Histone H2AX is a tumor suppressor protein that plays an important role in apoptosis. However, the mechanism underlying the association of H2AX with apoptosis in cancer cells remains elusive. Here, we showed that H2AX knockdown in lung cancer A549 cells affected the expression of 16 microRNAs (miRNAs), resulting in the downregulation of 1 and the upregulation of 15 miRNAs. MicroRNA-3196 (miR-3196) was identified as a target of H2AX and shown to inhibit apoptosis in lung cancer cells by targeting p53 upregulated modulator of apoptosis (PUMA). Phosphorylated H2AX (γH2AX) was shown to bind to the promoter of miR-3196 and regulate its expression. In addition, H2AX phosphorylation increased H3K27 trimethylation in the promoter region of miR-3196 and inhibited the binding of RNA polymerase II to the promoter of miR-3196, leading to the inhibition of miR-3196 transcription. Taken together, these results indicated that H2AX phosphorylation regulates apoptosis in lung cancer cells via the miR-3196/PUMA pathway.

## INTRODUCTION

Lung cancer, also known as lung carcinoma, is a malignant lung tumor characterized by uncontrolled cell growth in the tissues of the lung. The main primary types of lung cancer are small-cell lung carcinoma (SCLC) and non-small-cell lung carcinoma (NSCLC). NSCLC is the most common form of lung cancer and includes adenocarcinomas, squamous cell carcinomas, and large cell carcinomas. Lung cancer, which is the most common malignancy and the leading cause of cancer-related death, was responsible for 610,000 deaths in 2015 in China [1]. In contrast to the steady increase in survival rates for most cancers, the 5-year relative survival rate for lung cancer remains low [2]. Therefore, the identification of new candidate molecules involved in lung cancer is important to improve the diagnosis, prevention, and treatment of this disease.

Histone H2AX is a member of the histone H2A family and plays a critical role in the DNA damage repair response following induction of double-strand breaks (DSBs). The induction of DSBs by internal or external stresses triggers the accumulation of H2AX near the DNA breakage sites and its rapid phosphorylation at Ser139 on its C-terminal end. Thus, phosphorylated H2AX (γH2AX) is considered as a marker of DNA damage. In a previous study, we showed that H2AX can be phosphorylated by c-Jun N-terminal kinase (JNK) and its phosphorylation is required for DNA ladder formation [3]. In a follow-up study, we found that H2AX is also involved in the regulation of apoptosis in tumor cells [4]. However, the detailed mechanism underlying the association of H2AX with apoptosis in cancer cells remains elusive.

MicroRNAs (miRNAs) are small noncoding RNAs of approximately 22–25 nucleotides in length that play key roles in regulating the translation and degradation of mRNAs [5–7]. Certain miRNAs are deregulated in several types of cancer. An increasing amount of studies indicate that miRNAs may function as tumor suppressors or oncogenes and play a critical role in tumorigenesis and cancer progression. The differential expression of miRNAs in lung cancer and unaffected lung tissue provides valuable information for determining the prognosis of patients and predicting recurrence [8–11]. Previous studies focused on elucidating the mechanisms underlying the miRNA-mediated regulation of target genes; however, relatively little is known about the regulation of miRNA genes themselves.

In the present study, we used microarray assays to analyze the expression of miRNAs in lung cancer A549 cells in response to H2AX knockdown. We identified 1 downregulated miRNA and 15 unregulated miRNAs. Among the upregulated miRNAs, miR-3196 was shown to be involved in lung cancer cell apoptosis. miR-3196 inhibited lung cancer cell apoptosis by downregulating the pro-apoptotic protein p53 upregulated modulator of apoptosis (PUMA). In addition, we showed that γH2AX increases H3K27 trimethylation in the promoter region of miR-3196 and inhibits the binding of RNA polymerase II to the promoter of miR-3196. Cumulatively, our data indicates that H2AX phosphorylation regulates apoptosis in lung cancer cells through miR-3196/PUMA pathway.

## RESULTS

### H2AX phosphorylation negatively regulates miR-3196 expression

**Figure 1:**
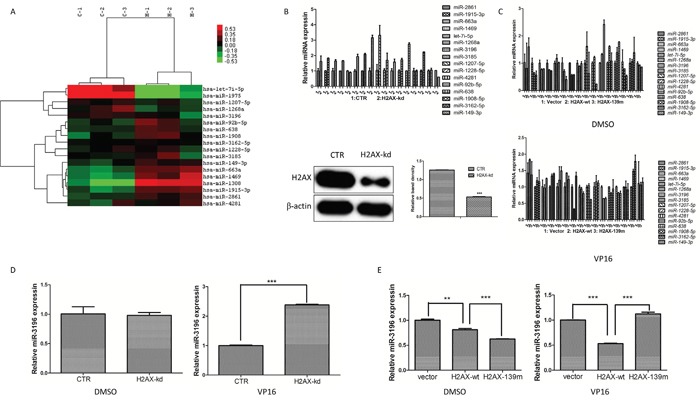
H2AX phosphorylation negatively regulates miR-3196 expression **A.** Differential expression of miRNAs in stable A549 control (CTR) cells (C-1, C-2 and C-3 for three biological replicates) and stable H2AX-knockdown A549 cells (H-1, H-2 and H-3 for three biological replicates) during apoptosis. Sixteen miRNAs were identified as significantly up- or down-regulated after VP16 treatment (100 μM) in H2AX-knockdown A549 cells compared with the CTR (*P <* 0.01). One miRNA was significantly downregulated and 15 miRNAs were significantly upregulated in H2AX knockdown cells as shown in the heat map. The top-right bar represents the signal levels of miRNA expression from -0.53 (green) to +0.53 (red). The individual identity of the significantly deregulated miRNAs is shown on the right border. **B.** The differentially expressed miRNAs regulated by H2AX were validated using qRT-PCR in A549 cells (CTR) and H2AX-knockdown A549 cells (H2AX-kd) after VP16 (100 μM) treatment (top panel). U6 was used as the internal control. The H2AX protein level in CTR and H2AX-kd cells was detected with western blotting (bottom panel). β-actin was used to confirm equal protein loading. **C.** Stable A549 cells transfected with H2AX-wt, H2AX-139m or vector were treated with DMSO (top panel) or VP16 (100 M, bottom panel) for 48 h and then the differentially expressed miRNAs were validated using qRT-PCR. U6 was used as the internal control. **D.** miR-3196 expression in CTR and H2AX-kd A549 cells after stimulation of apoptosis was detected by qRT-PCR. U6 was used as the internal control. **E.** Stable A549 cells transfected with H2AX-wt, H2AX-139m or vector were treated with VP16 (100 μM) for 48 h. miR-3196 expression was detected by qRT-PCR. U6 was used as the internal control. Error bars denote the mean ± SD. **P < 0.01; ***P < 0.001 by Student's t test.

To determine whether H2AX regulates miRNA expression in lung cancer cells during apoptosis, stable H2AX knockdown cells (A549) and control cells were treated with VP16, a topoisomerase II inhibitor to induce apoptosis. Analysis of differentially expressed miRNAs by microarray assay identified 16 deregulated apoptosis-related miRNAs (*P* < 0.01) in H2AX knockdown cells compared to control A549 cells. Of these 16 deregulated miRNAs, 1 was downregulated and 15 were significantly upregulated in H2AX knockdown cells (Figure 1A). The expression of these 16 miRNAs in H2AX-knockdown and control A549 cells was validated using quantitative RT-PCR (qRT-PCR), which showed that the expression of miRNAs was consistent with the microarray data (Figure 1B, upper panel). Additionally, the 16 miRNAs expression in A549 stable cell lines overexpressing H2AX-wt (wild type) or H2AX-139m (containing a mutation in Ser139 to block phosphorylation) were tested after VP16 treatment. The results indicated that only miR-3196 was significantly downregulated in stable H2AX-wt A549 cells, but was not changed in stable H2AX-139m A549 cells (Figure 1C). In our previous study, we revealed that VP16 induced H2AX phosphorylation at Ser139; we therefore confirmed in detail whether miR-3196 expression was really regulated by H2AX phosphorylation. For this purpose, the A549 stable cell lines overexpressing H2AX-wt, H2AX-139m and A549 stable H2AX-knockdown cells were induced with VP16, and the expression of miR-3196 was analyzed by qRT-PCR. The results showed that miR-3196 was significantly upregulated in stable H2AX-knockdown cells (Figure 1D), whereas it was significantly downregulated in stable H2AX-wt cells (Figure 1E). Furthermore, miR-3196 level was significantly higher in stable H2AX-139m cells treated by VP16 than in stable H2AX-wt A549 cells (Figure 1E). Taken together, these data demonstrated that H2AX phosphorylation at Ser139 regulates miR-3196 expression during apoptosis of lung cancer cells.

### miR-3196 decreases apoptosis in lung cancer cells

**Figure 2:**
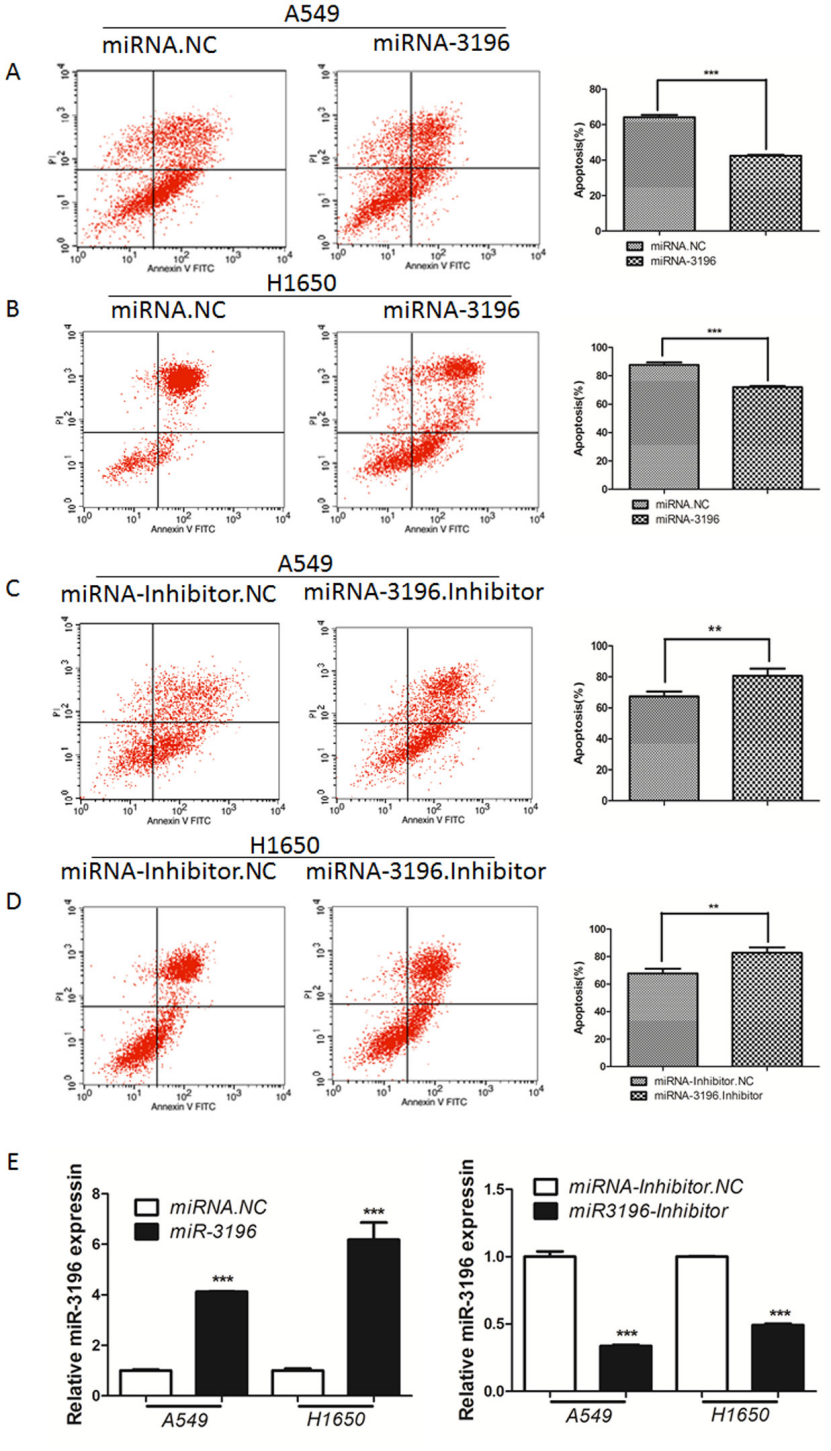
miR-3196 is involved in apoptosis in lung cancer cells **A.** Lung cancer A549 cells transfected with miR-3196 mimic (miR-3196) or negative control miRNA (miRNA.NC) for 24 h were induced with VP16 (100 μM) for 48 h and apoptotic cells were detected by flow cytometry (FCM). The histogram shows the percentage of apoptotic cells including early and late apoptotic cells. **B.** Lung cancer H1650 cells were transfected with miR-3196 mimic (miR-3196) for 24 h, followed by VP16 (100 μM) treatment for 48 h. The apoptotic cells were detected as in (A). **C.** A549 cells transfected with miR-3196 inhibitor or its negative control (miR-3196 inhibitor.NC) for 24 h were treated with VP16 (100 μM) for 48 h and the apoptotic cells were subjected to FCM analysis as in (A). **D.** H1650 cells were induced by VP16 (100 μM) after miR-3196 inhibitor or miRNA inhibitor-NC transfection for 24 h and the apoptotic cells were subjected to FCM analysis as in (A). **E.** Relative expression of miR-3196 was tested in A549 and H1650 cells after transfected with miR-3196 mimic (miR-3196) or miR-3196 inhibitor. U6 was used as the internal control. Error bars denote the mean ± SD. **P < 0.01, ***P < 0.001 by Student's t test.

Next, we tested whether miR-3196 modulates VP16-induced apoptosis in lung cancer cells. miR-3196 mimics or miR-3196 inhibitor were transfected into lung cancer A549 and H1650 cells, followed by treatment with VP16 for 48 h and detection of the number of apoptotic cells by flow cytometry (FCM). As shown in Figure 2, overexpression of miR-3196 significantly inhibited VP16-induced cellular apoptosis compared with control miRNA, decreasing the percentage of apoptotic A549 cells from 65% to 40% and that of apoptotic H1650 cells from 80% to 70% (Figure 2A and 2B, right panels). However, overexpression of a miR-3196 inhibitor significantly promoted VP16-induced apoptosis, resulting in an increase in the percentage of apoptotic A549 cells and H1650 cells from approximately 68% to 80% (Figure 2C and 2D, right panels). We also tested the miR-3196 expression after transfected with miR-3196 mimics or inhibitor. The results showed that miR-3196 expression was increased about 4-6 fold change after transfected with miR-3196 mimics in A549 or H1650 cells (Figure 2E, left panel), and transfection of miR-3196 inhibitor decreased miR-3196 level about 66% in A549 cells and 50% in H1650 cells (Figure 2E, right panel). Overall, these data provide strong evidence that miR-3196 decreased VP16-induced apoptosis in lung cancer cells.

### PUMA is a direct functional target of miR-3196

**Figure 3:**
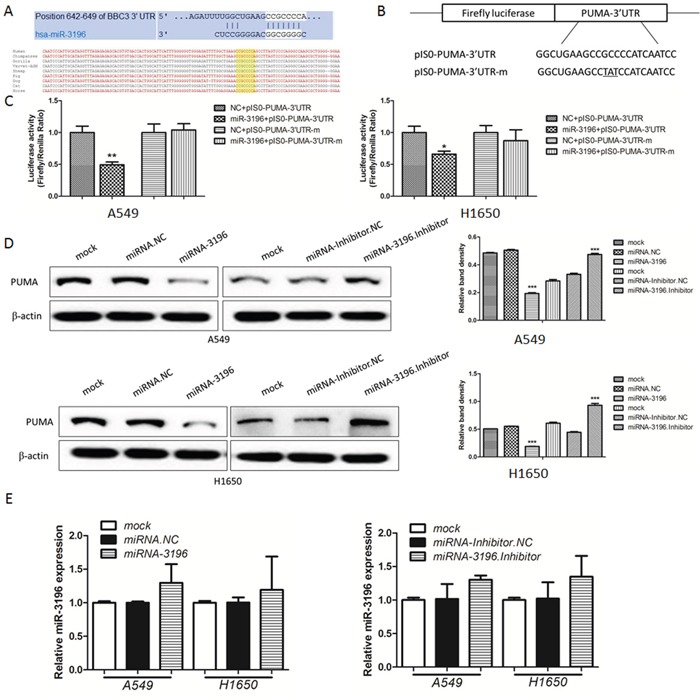
PUMA is a direct functional target of miR-3196 **A.** miR-3196 targeting site residues at nucleotides 642–649 of the *PUMA*-3′-UTR. Upper panel: sequence alignment of miR-3196 with binding sites on the *PUMA*-3′-UTR. Lower panel: sequences of the miR-3196 binding site within the *PUMA*-3′-UTR of nine species. **B.** Diagram of the luciferase reporter plasmids, including a plasmid with the full-length *PUMA*-3′-UTR insert (pIS0-PUMA-3′-UTR) and a plasmid with a mutant *PUMA*-3′-UTR (pIS0-PUMA-3′-UTR-m), which carried a substitution of three nucleotides within the miR-3196 binding site. **C.** A549 (left panel) and H1650 (right panel) cells were transfected with miR-3196 mimics (20 nM) combined with pIS0-PUMA-3′-UTR or pIS0-PUMA-3′-UTR-m. NC: negative control miRNA (miRNA.NC). pRL-SV40 Renilla was used for normalization of transfection efficiency. After 48 h, the luciferase activities were measured. **D.** Western blotting was used to detect PUMA protein after transfection of miR-3196 mimics (20 nM) or miR-3196 inhibitor (40 nM) in A549 (upper panel) or H1650 (bottom panel) cells. β-actin was detected as a loading control. **E.** *PUMA* mRNA in A549 and H1650 cell lines treated as in (D) was measured by qRT-PCR. β-actin was used as the internal control. *P < 0.05; **P < 0.01.

To elucidate the biological mechanisms underlying the effect of miR-3196 on the apoptosis of lung cancer cells, we investigated the potential targets of miR-3196. The target prediction programs miRanda and TargetScan (www.microrna.org and www.targetscan.org) were used to identify putative miR-3196 target genes. *PUMA* was identified as a target gene and found to bear a miR-3196 binding site in the 3′-untranslated region (3′-UTR), as shown in Figure 3A (upper panel). Comparison of the human sequence for interspecies homology revealed that the miR-3196 target sequence at nucleotides 642–649 of the *PUMA*-3′-UTR is highly conserved among nine species (Figure 3A, bottom panel). To verify this finding, the wild-type or mutant *PUMA*-3′-UTR was cloned downstream of the luciferase reporter gene in a pIS0-control vector, generating the pIS0-PUMA-3′-UTR and pIS0-PUMA-3′-UTR-m vectors (Figure 3B). Cotransfection of A549 (Figure 3C, left panel) or H1650 cells (Figure 3C, right panel) with miR-3196 and pIS0-PUMA-3′-UTR markedly reduced luciferase activity compared with the negative control (NC). By contrast, luciferase activity remained unchanged after cotransfection of A549 and H1650 cells with miR-3196 and pIS0-PUMA-3′-UTR-m (Figure 3C). These data indicated that miR-3196 directly targets the *PUMA* 3′-UTR. To further investigate whether miR-3196 regulates endogenous PUMA protein levels, miR-3196 mimics or inhibitor was transfected into A549 or H1650 cells and PUMA protein expression was assessed by western blotting. The results showed that miR-3196 overexpression significantly downregulated PUMA in both A549 and H1650 cells (Figure 3D, left panels). Conversely, PUMA was markedly upregulated in A549 and H1650 cells transfected with the miR-3196 inhibitor (Figure 3D, left panels). No changes in *PUMA* mRNA levels were detected (Figure 3E). Taken together, these results suggested that miR-3196 downregulates PUMA expression at the translational level.

### miR-3196 inhibits lung cancer cells apoptosis by downregulating PUMA

**Figure 4:**
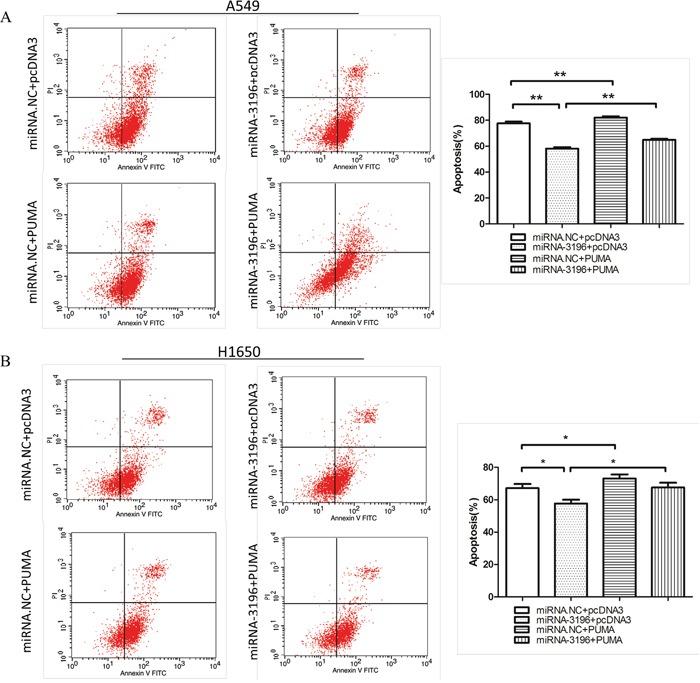
miR-3196 inhibits VP-16 induced apoptosis by downregulating PUMA **A.** A549 cells were transfected with miR-3196 mimics (miR-3196) and/or combined with pcDNA3-PUMA (PUMA) for 24 h, followed by treatment with VP16 (100 μM) for 48 h. The apoptotic cells were detected by FCM (left panel). Error bars denote the mean ± SD (right panel). **B.** H1650 cells were transfected and treated as in (A). The apoptotic cells were detected by FCM (left panel). Error bars denote the mean ± SD (right panel). * *P* < 0.05; **P < 0.01.

To determine whether the downregulation of PUMA by miR-3196 regulates lung cancer cell apoptosis, miR-3196 mimics and a PUMA expression plasmid (pcDNA3-PUMA) were co-transfected into A549 and H1650 cells. After 48 h of transfection, the cells were treated with VP16 (100 μM), and apoptotic cells were examined by FCM. Transfection with miR-3196 reduced VP-16-induced apoptosis compared with the control cells transfected with control miRNA, whereas PUMA overexpression increased the number of apoptotic cells compared with the controls transfected with vector alone (Figures 4A and 4B). However, cotransfection with pcDNA3-PUMA and miR-3196 reversed the effects of miR-3196 and PUMA overexpression on apoptosis in lung cancer cells. These results suggested that miR-3196 inhibits VP-16 induced apoptosis, at least in part, through the modulation of PUMA expression.

### γH2AX binds to the miR-3196 promoter and regulates PUMA expression

**Figure 5:**
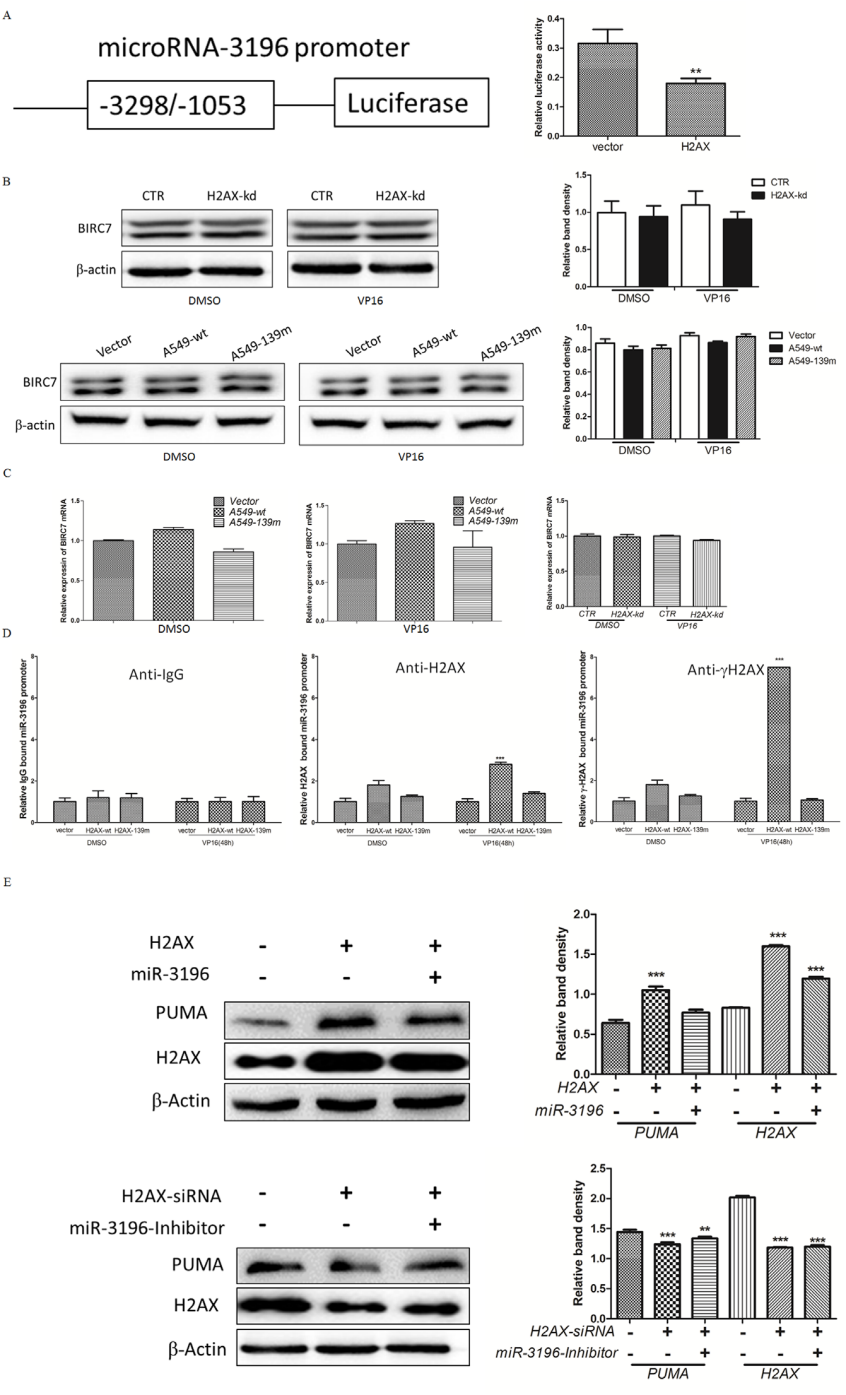
γH2AX binds to the promoter of the miR-3196 gene and regulates PUMA expression **A.** Schematic diagram of the miR-3196 gene promoter (left panel). The reporter gene plasmid was cotransfected with H2AX or control (vector) into A549 cells and luciferase activity was measured at 48 h after transfection (right panel). pRL-TK Renilla was used as an internal control. Values were normalized to Renilla luciferase activity.** *P* < 0.01. **B.** Expression of BIRC7 protein level in H2AX knockdown (upper panel) or overexpression (bottom panel) stable cells after VP-16 (100 μM) induction was evaluated by western blotting. **C**. BIRC7 mRNA level in H2AX knockdown (right panel) or overexpression (left and middle panels) stable cells treated as in (B) was detected by qRT-PCR. **D**. ChIPs were performed with IgG (served as a control), anti-H2AX and anti-γH2AX on H2AX-wt or H2AX-139m stable A549 cells treated with VP16 (100 μM) for 48 h. H2AX or γH2AX-associated promoter DNA amounts for miR-3196 were assessed by qRT-PCR with primer pairs flanking the promoter region of the miR-3196 promoter. *** *P* < 0.001. **E**. A549 cells were transfected with H2AX alone or combined with miR-3196 mimics (upper panel) for 48 h and PUMA expression was detected by western blotting. A549 cells were transfected with H2AX-siRNA alone or combined with miR-3196-inhibitor (lower panel) for 48 h, and PUMA expression was detected by western blotting. β-actin was detected as a loading control. ** *P* < 0.01, *** *P* < 0.001.

Since miR-3196 was identified as a highly upregulated miRNA in VP16-treated H2AX-knockdown cells (Figure 1C) and it inhibited VP-16-induced apoptosis by downregulating PUMA (Figure 4), we investigated whether miR-3196 expression is directly regulated by H2AX. miR-3196 is an intergenic miRNA located in the *BIRC7* gene and its promoter is unknown; therefore, we cloned the promoter of the *BIRC7* gene [12] into the pGL3-enhancer vector carrying the luciferase reporter gene (Figure 5A, left panel). A luciferase reporter assay revealed that miR-3196 promoter activity was decreased by H2AX overexpression in response to VP-16 treatment (Figure 5A, right panel). To exclude this possibility that H2AX maybe also regulate the BIRC7 gene expression also, which contributes to apoptotic inhibition by miR-3196, we detected the mRNA and protein level of BIRC7 upon H2AX knockdown/overexpression. The results showed that the expression of BIRC7 was not changed upon H2AX knockdown/overexpression (Figures 5B and 5C). To determine whether H2AX regulates miR-3196 by direct binding to its promoter, a chromatin immunoprecipitation (ChIP) assay was performed on extracts from H2AX-wt or H2AX-139m stable A549 cells to demonstrate the recruitment of H2AX or γH2AX to the miR-3196 promoter. The results showed a strong enrichment of γH2AX on the miR-3196 promoter after VP-16 treatment (Figure 5D). Blocking the phosphorylation of Ser139 of H2AX by overexpression of H2AX-139m inhibited the binding of H2AX to the miR-3196 promoter (Figure 5D). These data suggested that H2AX phosphorylation at Ser139 is required for binding to the miR-3196 promoter. To determine whether H2AX regulates PUMA expression through miR-3196, A549 cells were transfected with H2AX alone or in combination with miR-3196 mimics. The results indicated that overexpression of H2AX upregulated PUMA expression, whereas co-expression of H2AX and miR-3196 downregulated PUMA expression induced by H2AX overexpression (Figure 5E, upper panel). In addition, H2AX-siRNA-mediated H2AX knockdown downregulated PUMA (Figure 5E, bottom panels). However, co-transfection of H2AX-siRNA and miR-3196 inhibitor into A549 cells partially upregulated PUMA compared with transfection of H2AX-siRNA alone, suggesting that the miR-3196 inhibitor reduced the inhibiting effect of H2AX knockdown on PUMA expression (Figure 5E, bottom panel).

### H2AX phosphorylation increases H3K27 trimethylation in the promoter region of miR-3196 and inhibits RNA polymerase II binding to the miR-3196 promoter

**Figure 6:**
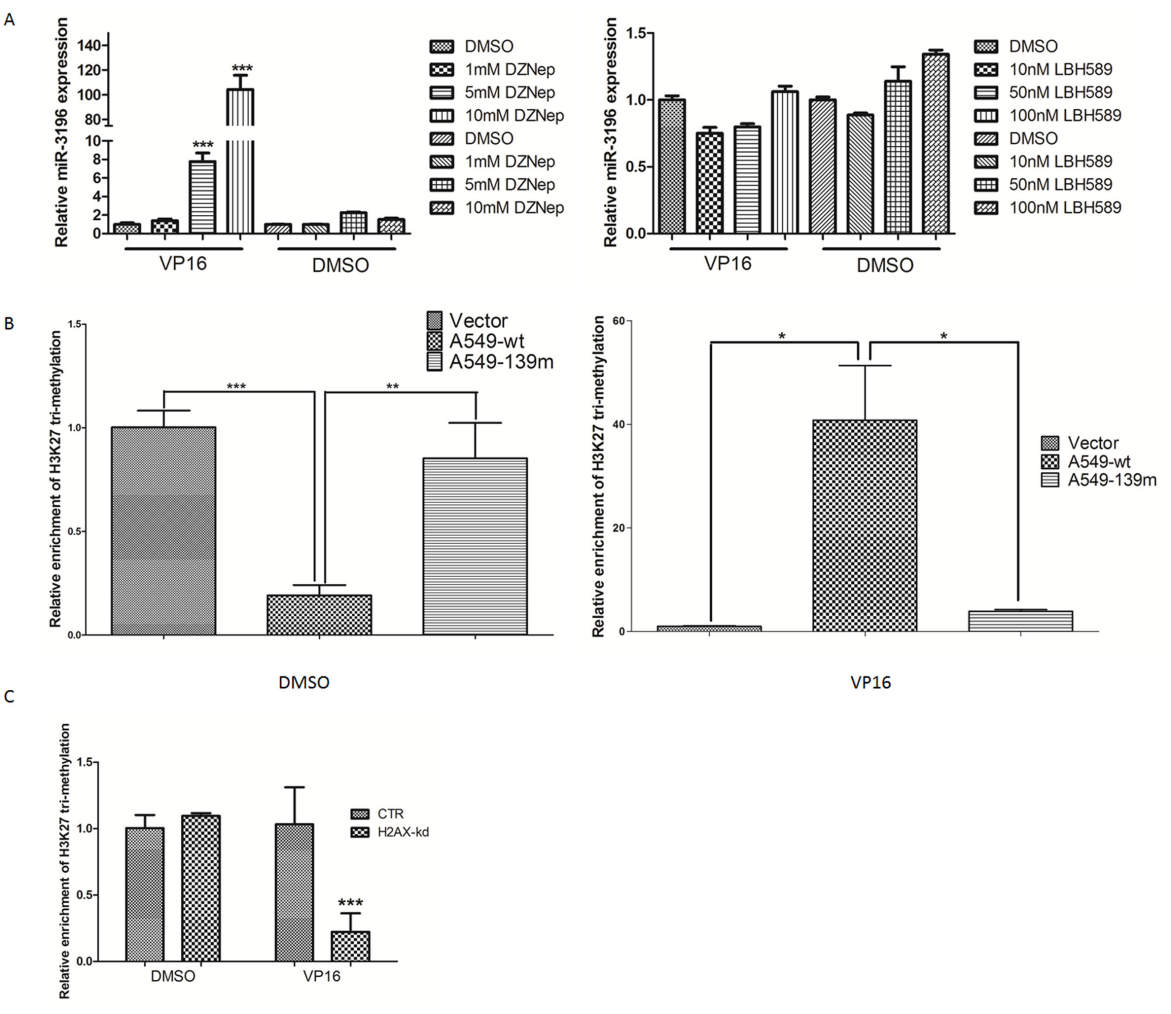
H2AX phosphorylation increases H3K27 trimethylation in the promoter region of miR-3196 **A.** A549 cells were treated with different concentrations of histone methyltransferase inhibitor (DZNep) (left panel) or histone deacetylase inhibitor (LBH589) (right panel), and the expression of miR-3196 was assessed by qRT-PCR. DMSO treatment served as a control. U6 was used as the internal control. **B.** Chromatin was immunoprecipitated from stable A549 control cells, H2AX-wt, or H2AX-139m cells using a H3K27 trimethylation antibody or a control IgG, and the enriched genomic fragment was amplified by qRT-PCR using primers located at positions -1963 – -1753 of the promoter region of miR-3196. **C.** Chromatin was immunoprecipitated from stable A549 control or A549 H2AX-knockdown cells (H2AX-kd) using a H3K27 trimethylation antibody or a control IgG, and the enriched genomic fragment was amplified as in (B). *P < 0.05, ** *P* < 0.01, *** *P* < 0.001.

**Figure 7:**
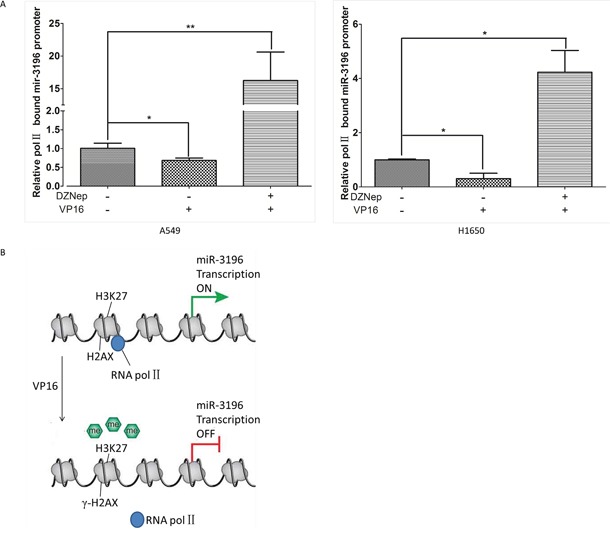
H2AX phosphorylation inhibits RNA polymerase II binding to the miR-3196 promoter **A.** A549 and H1650 cells were treated with histone methyltransferase inhibitor (DZNep 10 mM) and/or VP16 (100 mM). Chromatin was immunoprecipitated using an antibody against RNA polymerase II or IgG as a control, and the enriched genomic miR-3196 promoter fragment was amplified using primers located at positions -1963 – -1753 of the promoter region. *P < 0.05, ** *P* < 0.01. **B.** A model of miR-3196 transcription regulated by γH2AX.

We showed that γH2AX could bind the miR-3196 promoter and regulate PUMA expression through miR-3196. However, the mechanism by which γH2AX regulates miR-3196 expression remains unknown. Previous studies showed that γH2AX could increase histone H3 acetylation [13] and is associated with histone H4 methylation [14]. We speculated that γH2AX may regulate miR-3196 expression through histone methylation or acetylation in the promoter of miR-3196. A549 cells were treated with a histone methyltransferase inhibitor (DZNep) or histone deacetylase inhibitor (LBH589), and the expression of miR-3196 was assessed by qRT-PCR. The results showed that miR-3196 was upregulated by DZNep treatment (Figure 6A, left panel) but not by LBH589 treatment (Figure 6A, right panel), suggesting that miR-3196 transcription is regulated by histone methylation. Next, we checked H3K27 trimethylation on the miR-3196 promoter and found that the H3K27 trimethylation level of the miR-3196 promoter was significantly increased in A549 H2AX-wt stable cells after VP-16 treatment, whereas it was decreased in A549 H2AX-139m (Figure 6B) and H2AX-knockdown (Figure 6C) stable cells. Overall, these data demonstrated that H2AX phosphorylation increases H3K27 trimethylation on the miR-3196 promoter. To investigate whether H3K27 trimethylation affects the binding of RNA polymerase II to the miR-3196 promoter, we used DZNep to block histone methyltransferase and detected RNA polymerase II binding to the miR-3196 promoter. The results showed that DZNep treatment strongly increased the binding of RNA polymerase II to the miR-3196 promoter in A549 (Figure 7A, left panel) and H1650 cells (Figure 7A, right panel). Taken together, our data clearly demonstrated that H2AX phosphorylation increases H3K27 trimethylation in the promoter region of miR-3196 and affects the binding of RNA polymerase II to the miR-3196 promoter, leading to the inhibition of miR-3196 transcription (Figure 7B).

## DISCUSSION

Most miRNA genes are transcribed by RNA polymerase II in genomes either as independent transcriptional units with their own promoters or directly dependent on host gene transcription. Because miR-3196 gene is located in the *BIRC7* gene, we cloned the promoter of the *BIRC7* gene into the pGL3-enhancer vector and detected the effect of H2AX on the activity of the miR-3196 promoter using a luciferase reporter assay. Our results showed that H2AX overexpression dramatically inhibited miR-3196 promoter activity compared with that in the control cells without H2AX overexpression (Figure 5B), suggesting that H2AX negatively regulates miR-3196.

miR-3196 is a novel miRNA and its role in cancer is not clear. Previous studies showed that miR-3196 is downregulated in basal cell carcinoma [15] and in breast cancer patients with lymph node metastasis [16], whereas it is upregulated in papillary thyroid carcinoma patients with lung metastases [17]. miR-3196 was reported to be also associated with gastric cardia adenocarcinoma differentiation [18]. In the present study, we showed that miR-3196 is involved in apoptosis in lung cancer cells and inhibits VP-16 induced apoptosis by downregulating PUMA.

It is known that DNA damage can activate ataxia telangiectasia mutated protein (ATM), ataxia telangiectasia and Rad3-related (ATR) and DNA-dependent protein kinase (DNA-PK), which in turn phosphorylate H2AX at Ser139 [19–21]. The phosphorylated H2AX (γH2AX) gathers at the sites of DNA double-strand breaks (DSBs) and regulates DNA damage repair [22]. Our previous study showed that H2AX phosphorylation is required for ultraviolet (UV) A-induced DNA ladder formation, and the JNK/H2AX pathway cooperates with the caspase-3/CAD pathway to regulate cellular apoptosis [3]. Another study showed that EYA, a protein tyrosine phosphatase, dephosphorylates 142 tyrosine in H2AX and regulates apoptosis and survival decisions [23]. However, accumulating evidences suggest additional functions of histone H2AX modification [24–26]. H2AX is required for p21-induced cell cycle arrest after replication stalling [27]. γH2AX induces changes of chromatin that inhibit the assembly of transcription complexes without heterochromatin formation [28]. In addition, recent studies showed that γH2AX is required for high mobility group protein-mediated transcription [29]. Here, we showed that γH2AX could bind to the promoter of miR-3196 and regulate the expression of the apoptotic protein PUMA through miR-3196. Further analysis of the mechanism by which γH2AX regulates miR-3196 gene expression revealed that H2AX phosphorylation increases H3K27 trimethylation in the promoter region of miR-3196 and inhibits RNA polymerase II binding to the miR-3196 promoter. Based on these data, we proposed a model to explain the regulation of apoptosis by the γH2AX/miR-3196 pathway (Figure 7B). In this model, H3K27 trimethylation at the miR-3196 promoter regulated by H2AX phosphorylation at Ser139 is a key step for H2AX-mediated apoptosis. In summary, we identified a novel function of H2AX in the regulation of apoptosis at the transcriptional level.

## MATERIALS AND METHODS

### Cell culture

A549 and NCI-H1650 (H1650) cells were maintained in RPMI 1640 supplemented with 10% fetal bovine serum, 100 units/mL streptomycin, and 100 units/mL penicillin. Cells were grown to 80% confluence and then serum-deprived for 12 h prior to etoposide (VP16) (Sigma–Aldrich, St Louis, MO, USA) stimulation. Stable H2AX knockdown A549 cells and stable control cells were passaged in our laboratory as described before [4].

### Lentivirus preparation

HIV-based lentiviral expression plasmids, 293Ta lentiviral packaging cells, and a Lenti-Pac HIV Expression Packaging Kit were obtained from GeneCopoeia (Rockville, MD, USA). At 48 h after seeding, the 293Ta packaging cells were transfected with lentiviral vectors encoding H2AX-wt (wild type), H2AX-139m (containing a mutation in Ser139 to block phosphorylation), or empty vector (vector) using a Lenti-Pac™ HIV Expression Packaging Kit according to the manufacturer's instructions. The lentiviruses were harvested by collecting the pseudovirus-containing culture medium at 48 h post-transfection. A549 cells were transduced with the harvested lentiviral particles in the presence of 5 μg/mL polybrene (Santa Cruz Biotechnology, Santa Cruz, CA, USA) and cultured in selection medium with 2 μg/mL puromycin for approximately 2 weeks to allow the cells to grow to 90% confluence.

### miRNA microarrays

Stable A549 cells with H2AX knockdown and control stable A549 cells (CTR) were treated with VP16 (Sigma–Aldrich) (100 μM) for 48 h after overnight serum starvation. Then, RNA was isolated using the TRIzol reagent and analyzed using GeneChip miRNA 2.0 Array (Affymetrix, Santa Clara, CA, USA). Three biological replicates were used for each stable cell line. According to the data, the random variance model (RVM) t test was used to filter the differentially expressed miRNAs. After analysis of significance and false discovery rate (FDR) analysis, differentially expressed miRNAs were selected according to the *p* value threshold [4]. The miRNA expression data was deposited in the NCBI Gene Expression Omnibus (GEO) and are accessible through GEO Series accession number GSE68233.

### Oligonucleotides, plasmids, and transfection

miR-3196 mimics and miR-3196 inhibitor were synthesized by Genepharma (Shanghai, China). The full-length 3′-UTR of PUMA was subcloned into the pIS0 luciferase plasmid [30] to generate pIS0-PUMA-3′-UTR. The mutant construct of PUMA-3′-UTR, named pIS0-PUMA-3′-UTR-m, which carried a substitution of three nucleotides within the core binding site of the PUMA-3′-UTR, was generated using mutant PCR primers. Lipofectamine 2000 (Life Technologies Corporation, Grand Island, NY, USA) was used for transfection of DNA plasmids and oligonucleotides according to the manufacturer's protocol.

### RNA extraction and qRT-PCR

Total RNA was extracted with the TRIzol reagent (Life Technologies Corporation). Reverse transcription was performed using the FastQuant RT Kit (with gDNase) (TianGen, Beijing, China) according to the manufacturer's protocol. miR-3196 was reverse-transcribed by the looped primer, which binds to six nucleotides at the 3′ end of the miR-3196 molecule. Reverse transcription of PUMA mRNA was performed according to the manufacturer's protocol. RT-PCR was performed using SuperRealPreMix Plus (TianGen) according to the manufacturer's recommendations. The U6 small nuclear RNA and β-actin mRNA were used as internal controls for miR-3196 and PUMA mRNA, respectively. All reactions were run in triplicate.

### Luciferase assay

To test whether miR-3196 directly targets PUMA mRNA at the 3′-UTR, cells were cultured in 96-well plates and transiently co-transfected with firefly luciferase reporter gene constructs and miR-3196 mimics using Lipofectamine 2000. After 48 h, luciferase activity was measured using a dual luciferase reporter assay system according to the manufacturer's protocol (Promega, Madison, WI, USA). The pRL-TK Renilla was used as an internal control.

For promoter-driven luciferase assays, cells were cotransfected with the miR-3196 promoter construct (pGL3-miR-3196-P) and H2AX (pcDNA3-H2AX) expression constructs or control plasmid. Firefly luciferase and Renilla luciferase activities were determined at 48 h after transfection using the Dual-Luciferase Reporter Assay System (Promega). Values were normalized to Renilla luciferase activity.

### Flow cytometry

Cells transfected with negative control or miR-3196 mimics/inhibtors for 24 h were starved overnight and then treated with VP16 (Sigma-Aldrich) for 48 h. The FCM assay was performed using the Annexin V FITC Apoptosis Detection Kit (BD Biosciences, San Jose, CA, USA) according to the manufacturer's instructions.

### Western blotting

Cellular proteins were extracted after treatment as described previously [31]. Clarified cell lysates were equalized for protein concentration using the BCA (Bicinchoninic Acid) protein assay. The protein samples were resolved by SDS-PAGE and processed by western blotting. Primary antibodies against H2AX, BIRC7, PUMA and β-actin (Cell Signaling Technology, Beverly, MA, USA, 4970) were used to detect the corresponding proteins.

### Chromatin immunoprecipitation (ChIP)

ChIP assays were performed using the Magna ChIP kit (Millipore, Temecula, CA, USA) following the manufacturer's instructions. The qRT-PCR primers for miR-3196 are listed in Supplementary Table S1.

### ChIP antibodies

Antibodies used for ChIP were as follows: anti-histone H2AX (Abcam, Cambridge, MA, USA, ab11175), γH2AX (Abcam, ab2893), histone H3 (tri methyl K27) (Abcam, ab6002), RNA polymerase II (Millipore, 05-263), normal rabbit IgG (Millipore, 12-370) or normal mouse IgG (Millipore, 401211).

### Statistical analyses

Data were presented as the mean ± SD from at least three independent experiments, and the Student's t test was performed using SPSS 17.0 software. A *P*-value of ≤0.05 was considered significant.

## SUPPLEMENTARY MATERIALS TABLE



## References

[1] Chen W, Zheng R, Baade PD, Zhang S, Zeng H, Bray F, Jemal A, Yu XQ, He J (2016). Cancer statistics in China, 2015. CA Cancer J Clin.

[2] Siegel RL, Miller KD, Jemal A (2016). Cancer statistics, 2016. CA Cancer J Clin.

[3] Lu C, Zhu F, Cho YY, Tang F, Zykova T, Ma WY, Bode AM, Dong Z (2006). Cell apoptosis: requirement of H2AX in DNA ladder formation, but not for the activation of caspase-3. Mol Cell.

[4] Lu C, Xiong M, Luo Y, Li J, Zhang Y, Dong Y, Zhu Y, Niu T, Wang Z, Duan L (2013). Genome-wide transcriptional analysis of apoptosis-related genes and pathways regulated by H2AX in lung cancer A549 cells. Apoptosis.

[5] Lee RC, Ambros V (2001). An extensive class of small RNAs in Caenorhabditis elegans. Science.

[6] Lau NC, Lim LP, Weinstein EG, Bartel DP (2001). An abundant class of tiny RNAs with probable regulatory roles in Caenorhabditis elegans. Science.

[7] Lagos-Quintana M, Rauhut R, Lendeckel W, Tuschl T (2001). Identification of novel genes coding for small expressed RNAs. Science.

[8] Boeri M, Verri C, Conte D, Roz L, Modena P, Facchinetti F, Calabro E, Croce CM, Pastorino U, Sozzi G (2011). MicroRNA signatures in tissues and plasma predict development and prognosis of computed tomography detected lung cancer. Proc Natl Acad Sci U S A.

[9] Hu Z, Chen X, Zhao Y, Tian T, Jin G, Shu Y, Chen Y, Xu L, Zen K, Zhang C, Shen H (2010). Serum microRNA signatures identified in a genome-wide serum microRNA expression profiling predict survival of non-small-cell lung cancer. J Clin Oncol.

[10] Patnaik SK, Kannisto E, Knudsen S, Yendamuri S (2010). Evaluation of microRNA expression profiles that may predict recurrence of localized stage I non-small cell lung cancer after surgical resection. Cancer Res.

[11] Yu SL, Chen HY, Chang GC, Chen CY, Chen HW, Singh S, Cheng CL, Yu CJ, Lee YC, Chen HS, Su TJ, Chiang CC, Li HN, Hong QS, Su HY, Chen CC (2008). MicroRNA signature predicts survival and relapse in lung cancer. Cancer Cell.

[12] Dynek JN, Chan SM, Liu J, Zha J, Fairbrother WJ, Vucic D (2008). Microphthalmia-associated transcription factor is a critical transcriptional regulator of melanoma inhibitor of apoptosis in melanomas. Cancer Res.

[13] Lee HS, Park JH, Kim SJ, Kwon SJ, Kwon J (2010). A cooperative activation loop among SWI/SNF, gamma-H2AX and H3 acetylation for DNA double-strand break repair. EMBO J.

[14] Pei H, Zhang L, Luo K, Qin Y, Chesi M, Fei F, Bergsagel PL, Wang L, You Z, Lou Z (2011). MMSET regulates histone H4K20 methylation and 53BP1 accumulation at DNA damage sites. Nature.

[15] Sand M, Skrygan M, Sand D, Georgas D, Hahn SA, Gambichler T, Altmeyer P, Bechara FG (2012). Expression of microRNAs in basal cell carcinoma. Br J Dermatol.

[16] Wang B, Li J, Sun M, Sun L, Zhang X (2014). miRNA expression in breast cancer varies with lymph node metastasis and other clinicopathologic features. IUBMB Life.

[17] Qiu ZL, Shen CT, Song HJ, Wei WJ, Luo QY (2015). Differential expression profiling of circulation microRNAs in PTC patients with non-131I and 131I-avid lungs metastases: a pilot study. Nucl Med Biol.

[18] Gao S, Zhou F, Zhao C, Ma Z, Jia R, Liang S, Zhang M, Zhu X, Zhang P, Wang L, Su F, Zhao J, Liu G, Peng B, Feng X (2016). Gastric cardia adenocarcinoma microRNA profiling in Chinese patients. Tumour Biol.

[19] Stiff T, Walker SA, Cerosaletti K, Goodarzi AA, Petermann E, Concannon P, O'Driscoll M, Jeggo PA (2006). ATR-dependent phosphorylation and activation of ATM in response to UV treatment or replication fork stalling. EMBO J.

[20] Takahashi A, Ohnishi T (2005). Does gammaH2AX foci formation depend on the presence of DNA double strand breaks?. Cancer Lett.

[21] Stiff T, O'Driscoll M, Rief N, Iwabuchi K, Lobrich M, Jeggo PA (2004). ATM and DNA-PK function redundantly to phosphorylate H2AX after exposure to ionizing radiation. Cancer Res.

[22] Rogakou EP, Boon C, Redon C, Bonner WM (1999). Megabase chromatin domains involved in DNA double-strand breaks in vivo. J Cell Biol.

[23] Cook PJ, Ju BG, Telese F, Wang X, Glass CK, Rosenfeld MG (2009). Tyrosine dephosphorylation of H2AX modulates apoptosis and survival decisions. Nature.

[24] Steinel NC, Lee BS, Tubbs AT, Bednarski JJ, Schulte E, Yang-Iott KS, Schatz DG, Sleckman BP, Bassing CH (2013). The ataxia telangiectasia mutated kinase controls Igkappa allelic exclusion by inhibiting secondary Vkappa-to-Jkappa rearrangements. J Exp Med.

[25] Turinetto V, Orlando L, Sanchez-Ripoll Y, Kumpfmueller B, Storm MP, Porcedda P, Minieri V, Saviozzi S, Accomasso L, Cibrario Rocchietti E, Moorwood K, Circosta P, Cignetti A, Welham MJ, Giachino C (2012). High basal gammaH2AX levels sustain self-renewal of mouse embryonic and induced pluripotent stem cells. Stem Cells.

[26] Economopoulou M, Langer HF, Celeste A, Orlova VV, Choi EY, Ma M, Vassilopoulos A, Callen E, Deng C, Bassing CH, Boehm M, Nussenzweig A, Chavakis T (2009). Histone H2AX is integral to hypoxia-driven neovascularization. Nat Med.

[27] Fragkos M, Jurvansuu J, Beard P (2009). H2AX is required for cell cycle arrest via the p53/p21 pathway. Mol Cell Biol.

[28] Solovjeva LV, Svetlova MP, Chagin VO, Tomilin NV (2007). Inhibition of transcription at radiation-induced nuclear foci of phosphorylated histone H2AX in mammalian cells. Chromosome Res.

[29] Singh I, Ozturk N, Cordero J, Mehta A, Hasan D, Cosentino C, Sebastian C, Kruger M, Looso M, Carraro G, Bellusci S, Seeger W, Braun T, Mostoslavsky R, Barreto G (2015). High mobility group protein-mediated transcription requires DNA damage marker gamma-H2AX. Cell Res.

[30] Yekta S, Shih IH, Bartel DP (2004). MicroRNA-directed cleavage of HOXB8 mRNA. Science.

[31] Dong Y, Xiong M, Duan L, Liu Z, Niu T, Luo Y, Wu X, Xu C, Lu C (2014). H2AX phosphorylation regulated by p38 is involved in Bim expression and apoptosis in chronic myelogenous leukemia cells induced by imatinib. Apoptosis.

