# Supplementary material for: MicroRNA-3196 is inhibited by H2AX phosphorylation and attenuates lung cancer cell apoptosis by downregulating PUMA
Source: Oncotarget. 2016 Oct 21;7(47):77764–76. doi: 10.18632/oncotarget.12794 (PMC5363619; doi:10.18632/oncotarget.12794)
Supplement: Supplementary file 1 [file oncotarget-07-77764-s001.pdf]

MicroRNA-3196 is inhibited by H2AX phosphorylation and attenuates lung cancer cell apoptosis by downregulating PUMA

SUPPLEMENTARY TABLE

Supplementary Table S1: Primers used in this study

**The primers for plasmid construction**	
miR-3196-F	ACACTCCAGCTGGG CGGGGCGGCAGG
miR-3196-R	CTCAACTGGTGTCGTGGAGTCGGCAATTCAGTTGAGGAGGCCCC
PUMA-clone-F	CTAGCTAGCATGGCCCGCGCACGCCAGGAG
PUMA-clone-R	GCTCTAGACTAATTGGGC TCCATCTCGGG
PUMA-realtime-F	TGCTGTAGATACCGGAATG
PUMA-realtime-R	TTCCACTGTTCCAATCTGAT
PUMA-UTR-F	TTGAGCTC CCTGCCTCACCTTCATCA
PUMA-UTR-R	GCTCTAGATTCCACTGTTCCAATCTGAT
PUMA-UTR-M-F	GGCTGAAGCCTATCCAGCCTTAG
PUMA-UTR-M-R	CTAAGGCTGGATAGGCTTCAGCC
miR-3196-p-F	GGAATTCGCTAGCACACCAACCTTGTCTCCTCTCCGA
miR-3196-p-F	CTCGAGGTTTCTCCACACGCATCGCAGTG
miR-3196-ChIP-F	CAGCTTGAGAGTTCAGAGCAA
miR-3196-ChIP-R	CGGTTACCTTTGGTCACTTCA
U6-F	CTCGCTTCGGCAGCACA
U6-R	AACGCTTCACGAATTTGCGT
miRNA URP	TGGTGTCGTGGAGTCG
